# Donkey Fascioliasis Within a One Health Control Action: Transmission Capacity, Field Epidemiology, and Reservoir Role in a Human Hyperendemic Area

**DOI:** 10.3389/fvets.2020.591384

**Published:** 2020-11-05

**Authors:** Santiago Mas-Coma, Paola Buchon, Ilra R. Funatsu, Rene Angles, Cristina Mas-Bargues, Patricio Artigas, M. Adela Valero, M. Dolores Bargues

**Affiliations:** ^1^Departamento de Parasitología, Facultad de Farmacia, Universidad de Valencia, Valencia, Spain; ^2^Unidad de Limnología, Instituto de Ecología, Universidad Mayor de San Andrés (UMSA), La Paz, Bolivia; ^3^Cátedra de Parasitología, Facultad de Medicina, Universidad Mayor de San Andrés (UMSA), La Paz, Bolivia; ^4^Departamento de Fisiología, Facultad de Medicina, Universidad de Valencia, Valencia, Spain

**Keywords:** human fascioliasis hyperendemic, One Health, donkey, *Fasciola hepatica*, *Galba truncatula* experimental transmission, field epidemiology, reservoir role, Bolivia

## Abstract

A One Health initiative has been implemented for fascioliasis control in a human hyperendemic area for the first time. The area selected for this multidisciplinary approach is the Northern Bolivian Altiplano, where the highest prevalences and intensities in humans have been reported. Within the strategic intervention axis of control activities concerning animal reservoirs, complete experimental studies, and field surveys have been performed to assess the fascioliasis transmission capacity and epidemiological role of the donkey for the first time. Laboratory studies with altiplanic donkey-infecting *Fasciola hepatica* and altiplanic *Galba truncatula* snail vector isolates demonstrate that the donkey assures the viability of the whole fasciolid life cycle. Several aspects indicate, however, that *F. hepatica* does not reach, in the donkey, the level of adaptation it shows in sheep and cattle in this high altitude hyperendemic area. This is illustrated by a few-day delay in egg embryonation, longer prepatent period despite similar miracidial infectivity and shorter patent period in the intramolluscan development, lower cercarial production per snail, different cercarial chronobiology, shorter snail survival after shedding end, shorter longevity of shedding snails, and lower metacercarial infectivity in Wistar rats. Thus, the role of the donkey in the disease transmission should be considered secondary. Field survey results proved that liver fluke prevalence and intensity in donkeys are similar to those of the main reservoirs sheep and cattle in this area. Fasciolid egg shedding by a donkey individual contributes to the environment contamination at a rate similar to sheep and cattle. In this endemic area, the pronounced lower number of donkeys when compared to sheep and cattle indicates that the epidemiological reservoir role of the donkey is also secondary. However, the donkey plays an important epidemiological role in the disease spread because of its use by Aymara inhabitants for good transport, movements, and travel from one locality/zone to another, a repercussion to be considered in the present geographical spread of fascioliasis in the Altiplano due to climate change. Donkey transport of parasite and vector, including movements inside the zone under control and potential introduction from outside that zone, poses a problem for the One Health initiative.

## Introduction

Human fascioliasis is a parasitic disease to which only secondary public health importance was given until the 1990s (1). The scenario completely changed henceforth, when the number of case reports began to gradually increase in both the Old and New Worlds, but mainly with the description of human endemic areas in many developing countries of Latin America, Africa, and Asia (2), although human infection also occurs in developed countries (3). The public health importance of this emergence was early on recognized by the World Health Organization when including this disease among the group of food-borne trematodiases within the list of Neglected Tropical Diseases (NTDs) (4).

Among the many factors underlying this decision of WHO, the following stand out: (i) the worldwide distribution of this disease caused by two large-sized liver fluke species, *Fasciola hepatica* and *F. gigantica* (5); (ii) its high pathogenicity (6–8), immunological consequences in both the acute (9) and chronic phases (10) of the disease, and its increasing morbidity caused by the immunesuppression-induced very frequent coinfections with other pathogenic microorganisms (11, 12) and parasites (13, 14); (iii) the strong influences of climate change and global change on its transmission and epidemiology (15, 16) because of the lack of a premunition buffer at life cycle end at definitive host level (17, 18); and (iv) its impact on the development of rural communities of low-income countries, including severe clinical pictures (8, 19) and sequelae (8, 20).

The availability of a very efficient drug for human treatment, triclabendazole (21), became crucial in this WHO's decision and subsequent defining of the worldwide strategy of preventive chemotherapy according to different control programs. Such control approaches were adapted to the different transmission patterns and epidemiological situations in the countries presenting human endemic areas and are to be strengthened within the new WHO 2030 road map on NTDs by contributing to sustainable and resilient health systems (22).

South America is the continent presenting the highest number of human fascioliasis endemic areas reported. These areas are characterized for their location at high altitudes in altiplanos and valleys of the Andean region. The main countries affected by this disease because of presenting human endemic areas are Peru (14, 23, 24), Bolivia (13, 25, 26), Argentina (27–29), and Chile (30, 31).

Bolivia stands out because of including the hyperendemic area where the highest prevalences and intensities of fascioliasis have been reported in humans (32) and where children become infected very early in their lives (33). This area is distributed throughout the Northern Bolivian Altiplano, between Lake Titicaca and the valley of the capital city La Paz, at 3,820–4,100 m a.s.l. In 2007–2008, a wide fascioliasis control initiative by yearly mass treatment campaigns was launched by the World Health Organization (WHO) in this area (34, 35) and has been yearly implemented henceforth. Subsequent interannual monitoring assessments indicated infection and reinfection of children by ingestion of metacercariae with freshwater plants or drinking water (36), as a consequence of the high endemicity maintained by the infection rates in livestock (32, 37).

At a PAHO-WHO meeting in La Paz in 2014, it was decided to implement a One Health action to decrease the human risk of liver fluke infection. The One Health is a strategy that encourages interdisciplinary collaboration and communication on health at the human-animal-environmental interface, including multidisciplinary efforts to attain optimal health of people and animals, and the most appropriate measures for the environment (38). This type of approach has been supported by WHO, FAO, and OIE to face the control of zoonotic diseases (39, 40). For the control of fascioliasis in the Bolivian Altiplano, the initiative was planned to include long-term experimental studies, field monitoring assessments and control activities, according to modern standards already analyzed for trematode diseases (41–43).

The wide heterogeneity of transmission patterns and epidemiological scenarios of fascioliasis creates difficulties when applying control activities within a One Health action. Two factors cause these problems:

- Snail vector specificity: Fasciolid species use a wide spectrum of freshwater species of the family Lymnaeidae which present different ecological requirements. These snails are distributed worldwide, but only given species groups are used by the liver flukes, mainly species of *Galba*/*Fossaria* by *F. hepatica*, species of the *Radix* group by *F. gigantica*, and a singular species *Pseudosuccinea columella* by both fasciolids (44). In South America, *Radix* is absent and fascioliasis transmission is assured by *Galba*/*Fossaria* species which are malacologically indifferentiable and need DNA marker sequencing for specimen classification (45, 46). Fortunately, only one lymnaeid species has been proved to inhabit the Northern Bolivian Altiplano hyperendemic area, namely *Galba truncatula* imported from Europe by the Spanish “conquistadores” some time ago and which is geographically spreading along the Altiplano at present, due to climate change and human activities (47).
- Mammal host specificity: The adult stage of fasciolids is able to successfully infect and develop in many different domestic and wild species of mainly herbivorous but also omnivorous mammals. In the Bolivian Altiplano, similarly as throughout all of the Americas, only *F. hepatica* is present, which pronouncedly simplifies the disease characteristics. Wild lagomorphs and rodents, including domestic guinea pigs (*Cavia porcellus*, locally known as “cuyes” or “quwis”), were already proved to play no role in fascioliasis transmission (48). Among domestic animals, only sheep, cattle, pigs, and donkeys have been involved as important reservoirs in that area (32). Other species such as goats, horses, llamas and alpacas, despite being present, play no transmission role due to different reasons (49).

Within the aforementioned One Health initiative, the aim of the present study is to expose the results obtained in experimental studies on *F. hepatica* transmission and field surveys on animal liver fluke infection to assess the role of the donkey in the transmission and epidemiology of the disease in the very high altitude hyperendemic area of the Northern Bolivian Altiplano. To catalog the contribution of the donkey to fascioliasis in this area, results are compared with those of similar studies previously performed on the main altiplanic reservoir species: sheep and cattle (50).

The laboratory studies included the experimental follow-up of the development stages of egg, miracidium, lymnaeid snail vector infection, intramolluscan larval development, cercarial production, chronobiology of the cercarial shedding, vector survival to infection, metacercarial infectivity of mammal host, and adult stage development of altiplanic donkey isolates through altiplanic *G. truncatula* isolates. This is the first time that such experimental fascioliasis studies focus on the donkey. The field studies included the assessment of prevalence, intensity, egg measurements, and egg shedding rates in nature. This is the first time that the epidemiological role of the donkey as animal reservoir is assessed in a human endemic area.

## Materials and Methods

### Experimental Studies

#### Fasciolid Materials

**Figure 1:**
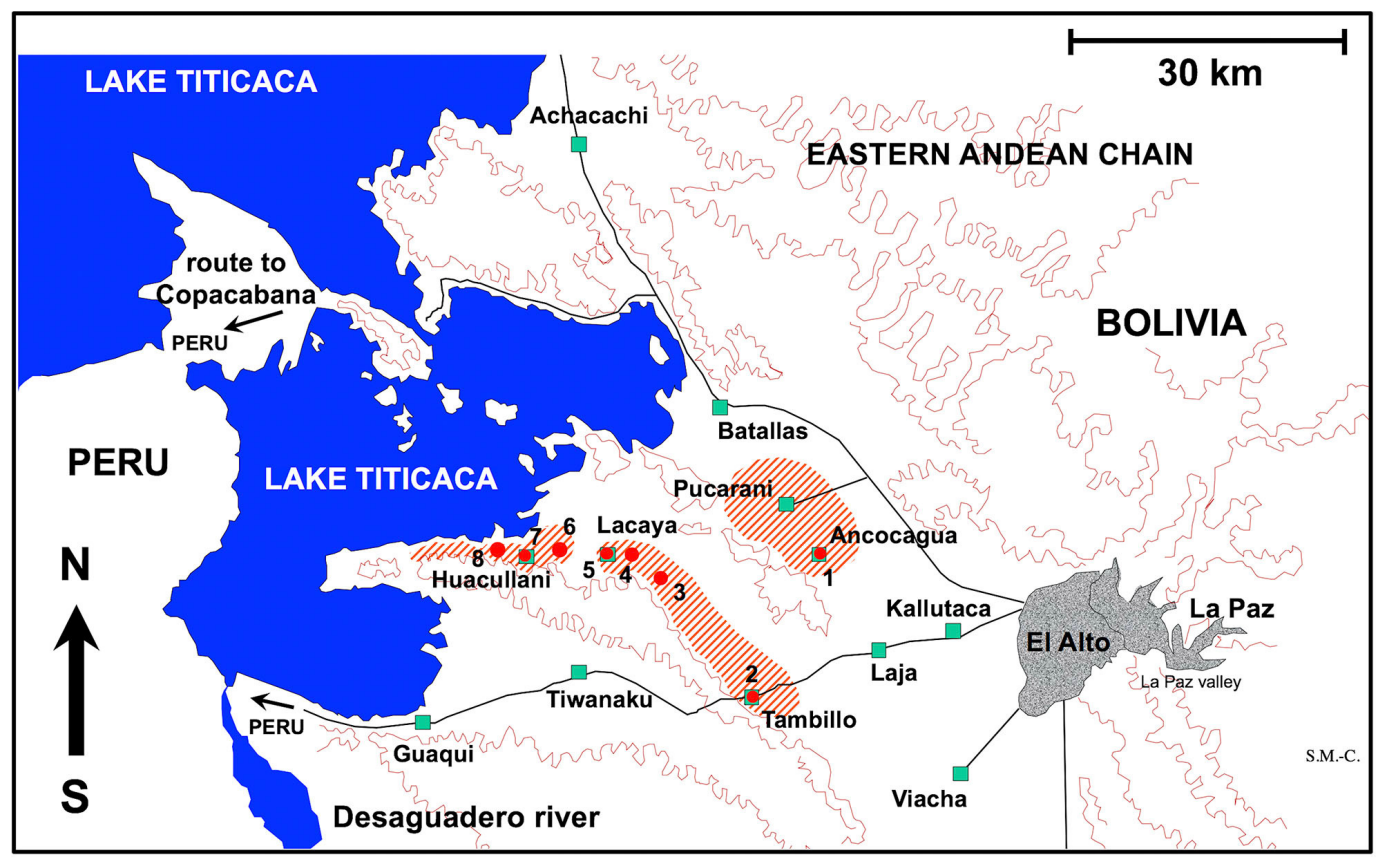
Map showing the Northern Bolivian Altiplano human fascioliasis hyperendemic area, at 3,820–4,100 m altitude, including zones where donkeys were surveyed and localities where lymnaeid snail vector specimens of *Galba truncatula* were collected. Localities: (1) Ancocagua; (2) Tambillo; (3) Korila; (4) Chiripujo; (5) Lacaya Baja; (6) Chojasihui; (7) Huacullani; (8) Queroni.

Fecal samples from naturally infected donkey individuals from the Altiplanic locality of Ancocagua were used for the experimental study of the embryonation of the eggs of *F. hepatica* (Figure 1). Eggs isolated by filtration were conserved in natural water under complete darkness at 4°C until starting of the embryogenesis follow up study.

For the experimental infection of lymnaeid snails, *F. hepatica* eggs were similarly obtained from donkeys from Ancocagua (Figure 1). Eggs were similarly isolated by filtration and conserved until used for snail infection in the laboratory.

For the experimental infection of Wistar rats, *F. hepatica* metacercariae were obtained from the aforementioned experimentally infected lymnaeid snails. These metacercariae were stored in natural water in total darkness until required. The storage temperature was 4°C, according to the usual standards in liver fluke studies (51).

Eggs of *F. hepatica* were also collected from sheep, cattle, and pigs of the same Northern Bolivian Altiplano human hyperendemic area, although the studies of the isolates of these three definitive reservoir hosts are the focus of other articles to avoid excessive text length and reference number.

#### Study of the Egg Embryogenesis

**Figure 2:**
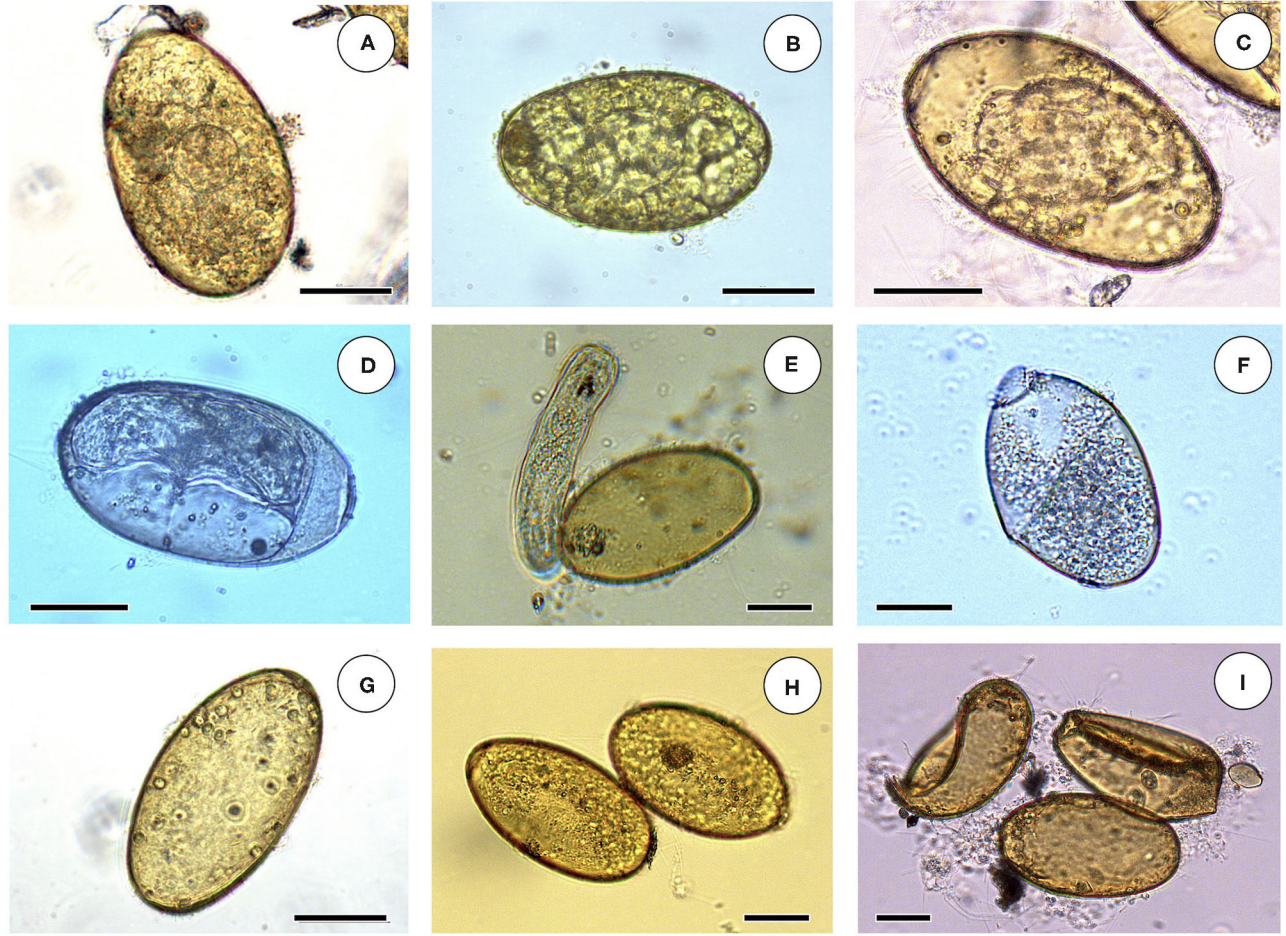
Embryonation stages in liver fluke eggs: **(A)** egg including an early developing morula (E.E.D.M.); **(B)** egg in the phase of advanced morula, showing vitelline granules and spheroidal cells (E.A.M.); **(C)** egg in the phase of outlined miracidium, in which a miracidial form begins to be observed (E.O.M.); **(D)** egg in the phase of developed miracidium, in which a fully developed miracidium is observed inside (E.D.M.); **(E)** empty egg immediately after miracidium hatching; **(F)** broken open egg after miracidial release; **(G)** empty egg; **(H)** two degenerated eggs; **(I)** two broken eggs and an empty egg. Scale bar = 40 μm.

The egg embryogenesis was experimentally followed at constant 20°C, at microscopic study intervals of 4 days. Egg development was made by differentiation of (i) eggs including an early developing morula (E.E.D.M.), (ii) eggs in the phase of advanced morula, showing vitelline granules and/or spheroidal cells (E.A.M.), (iii) eggs in the phase of outlined miracidium, in which a miracidial form begins to be observed (E.O.M.), and (iv) eggs in the phase of developed miracidium, in which a fully developed miracidium is observed inside (E.D.M.). For each 4-day study, a total of 33 eggs from each of four host individuals were analyzed between slip and coverslip. Counting did not only include E.E.D.M., E.A.M., E.O.M., and E.D.M., but also (i) degenerated eggs, (ii) empty eggs, and (iii) broken eggs (Figure 2). Degenerated, empty, and broken eggs (eggs easily break when open and empty after miracidial release) are very few at the beginning but of course increase with time and are the marked majority in the last days of the follow-up study. Egg counts were noted in percentages per observational day. Percentages of degenerated, empty and broken eggs are not included in the graph of follow-up curves because they do intervene in the transmission.

#### Experimental Infection of Snails

Fully embryonated eggs were put under light to force the hatching of developed miracidia which were afterwards used for the experimental infection of snails (52). Only laboratory-borne snails were used. Lymnaeid snails of a size of 4.0–5.0 mm were used to assess infection susceptibility, by exposing each snail to miracidia for 4 h in a small Petri dish containing 2 ml of fresh water. The disappearance of the miracidium was taken as verification of its successful penetration into the snail.

The donkey *F. hepatica* isolate was used for individual infection assays of lymnaeid snails under the experimental conditions of a monomiracidial dose and 20/20°C day/night temperature according to a photoperiod of 12 h light/12 h darkness in climatic chambers (HPS-1500, VB-0714, and HPS-500 models of Heraeus-Vötsch) (46). The characteristics, conditions, and number of snails in these snail infection experiments are detailed in Table 1. After the infection, snails were returned to 2,000 ml containers, at 90% relative humidity (r.h.), 20/20°C day/night temperature according to 12/12 h light/darkness, and dry lettuce *ad libitum*, until day 30 post-infection, in which they were again isolated in Petri dishes to allow daily monitoring of cercarial shedding by individual snails. Lettuce was provided *ad libitum* to each snail in a Petri dish during both shedding and post-shedding periods until death of the snail. The cercarial shedding was followed by daily counting of metacercariae in each Petri dish.

**Table 1:** Experimental infections of altiplanic lymnaeid snails with *Fasciola hepatica* donkey isolate from the Northern Bolivian Altiplano human hyperendemic area.

**Host isolate**	**Donkey**	**Sheep^a^**	**Cattle^a^**
*F. hepatica* geogr. origin	Ancocagua	Batallas	Batallas
Lymnaeid geogr. origin	Tambillo	Huacullani	Huacullani
Miracidial dose	Mono-miracidial	Mono-miracidial	Mono-miracidial
Temperature (12 day/12 h night)	20/20°C	20/20°C	20/20°C
No. lymnaeids infected	35	62	55
No. survivor snails at beginning of shedding (%)	25 (71.4%)	54 (87.1%)	48 (87.3%)
No. shedding snails (%)	7 (28.0%)	28 (51.8%)	12 (25.0%)
Prepatent period in dpi (mean)	52–69 (60.5)	48–92 (55.6)	49–76 (55.5)
Shedding end in dpi (mean)	52–117 (86.1)	52–136 (89.4)	58–135 (101.6)
Shedding length in days	1–49 (26.6)	1–88 (34.7)	1–85 (47.1)
No. total cercariae shed	333	5,542	3,672
No. cercariae/snail (mean)	2–92 (47.6)	8–562 (197.9)	8–581 (306.0)
Snail survival after shedding end in days	1–43 (14.8)	1–132 (24.5)	1–133 (42.3)
Longevity of shedding snails in dpi	77–126 (101.0)	53–192 (113.8)	76–268 (143.9)
Longevity of non-shedding snails in dpi	34–183 (60.7)	49–196 (139.1)	31–209 (105.4)

*dpi, days post-infection*.

a *Data from Mas-Coma et al. (50)*.

#### Laboratory Cultures of the Snail Vector

*Galba truncatula* has recently been proved to be the only lymnaeid species inhabiting the Northern Bolivian Altiplano hyperendemic area, by the sequencing of complete nuclear ribosomal DNA and mitochondrial DNA markers (47). This species is of European origin and differs from the Neotropical species of the *Galba*/*Fossaria* group of lymnaeids which also act as vectors of fascioliasis in South America (45). Living specimens of *G. truncatula* were collected in the locality of Tambillo (Figure 1) and transported under isothermal conditions for their laboratory adaptation to standardized controlled conditions of 20°C, 90% r.h. and a 12/12 h light/darkness photoperiod in the aforementioned precision climatic chambers. The possible natural infection by fasciolids was always individually verified prior to the launch of laboratory cultures. This was performed by keeping each lymnaeid specimen isolated in a Petri dish containing a small amount of natural water. After 24 h, the presence or absence of motionless metacercarial cysts or moving cercariae was verified in each Petri dish. Non-infected lymnaeids were arranged in standard breeding boxes containing 2,000 ml fresh water, to assure locality-pure cultures. The water was changed weekly and lettuce added *ad libitum*.

#### Laboratory Infections of Wistar Rats

A total of 18 male Wistar rats (Iffa Credo, Barcelona, Spain) aged 4–5 weeks were used throughout. A balanced commercial rodent diet (Panlab Chow A04) and water were provided *ad libitum*, according to standards previously reported (53).

Wistar rats were infected according to methods previously described (54). A dose of 20 *F. hepatica* metacercariae per rat was used (Table 2). Animal care, animal health, body condition, and well-being were assessed on a weekly basis by means of checking their body weight and the appearance of the fur. Infected animals presented a lower body weight than negative controls at the end of the experiment. No mortality occurred. Infection prevalence and intensity (number of worms successfully developed in each rat) were established by necropsy 12 weeks after infection. Metacercariae were inoculated orally by means of a gastric tube. The number of Wistar rats in each infection experiment are noted in Table 2. Finally, animals were euthanized with an overdose of an anesthetic (IsoFlo; Dr Esteve SA, Barcelona, Spain), and *F. hepatica* worms were collected under a dissecting microscope, according to methods already outlined before (55). The bile duct was initially examined for the presence of flukes, followed by the whole liver, although the rest of the organs were also evaluated. The thoracic and abdominal viscera and cavities were examined and thoroughly rinsed with water to assure the recovery of all worms.

**Table 2:** Experimental infections of Wistar rats with experimentally obtained metacercariae from donkey isolate and comparison with sheep and cattle isolates from the Northern Bolivian Altiplano human hyperendemic area.

**Host isolate**	**Donkey**		**Sheep^a^**		**Cattle^a^**	
*F. hepatica* geographical origin	Ancocagua	Ancocagua	Batallas	Ancocagua	Kallutaca	Batallas
Age of metacercariae	6 weeks	10 weeks	1 week	2 weeks	6 weeks	8 weeks
No. metacercariae inoculated per rat	20	20	20	20	20	20
No. inoculated rats	8	10	14	23	4	4
No. rats infected (%)	3 (37.5%)	4 (40.0%)	11 (78.6%)	18 (78.3%)	4 (100%)	2 (50.0%)
No. flukes recovered per rat (mean)	1–6 (3.3)	2–3 (2.7)	1–8 (3.6)	1–10 (3.7)	1–2 (1.7)	1–2 (1.5)
Intensity^b^	6.2%	5.5%	14.3%	14.6%	8.8%	3.7%
Mean % flukes recovered/rat^c^	16.7%	11.2%	18.2%	18.6%	8.8%	7.5%

a *Data from Mas-Coma et al. (50)*.

b *Intensity = total % of flukes recovered = (total No. of flukes recovered/total No. of metacercariae administered in all rats) × 100*.

c *Mean % flukes recovered/rat = Mean % of flukes recovered per infected rat = (flukes recovered/metacercariae administered per infected rat) × 100*.

### Field Surveys of Donkeys

#### Host Animals Studied

Fecal samples from a total of 84 donkeys were collected from different zones of the Northern Bolivian Altiplano hyperendemic area (Table 3). The geographical distribution of the localities and zones surveyed is shown in Figure 1.

**Table 3:** Prevalence and intensity of *Fasciola hepatica* infection found by coprological analyses in donkeys of different zones of the Northern Bolivian Altiplano human hyperendemic area and comparison with local prevalences in sheep and cattle from the same zones.

**Locality**	**Endemic zone**	**Donkey**				**Sheep^a^**	**Cattle^a^**
		**Prevalence**		**Intensity**		**Prevalence**	**Prevalence**
		**No. analyzed/infected**	**%**	**Range (epg)**	**Mean (epg)**	**No. analyzed/infected (%)**	**No. analyzed/infected (%)**
Ancocagua	Pucarani	35/3	8.6^b^	3–43	22.3	170/97 (57.1)	195/22 (11.3)
Tambillo	Tambillo-Lacaya	2/0	0^c^	–	–	230/113 (49.1)	50/2 (4.0)
Korila	Tambillo-Lacaya	1/0	0^c^	–	–	100/87 (87.0)	–
Chiripujo	Tambillo-Lacaya	2/0	0^c^	–	–	–	141/17 (12.0)
Lacaya Baja	Tambillo-Lacaya	10/3	30.0^b^	11–58	32.7	100/64 (64.0)	43/3 (47.0)
Chojasihui	Huacullani	6/3	50.0	14–101	50.7	100/70 (70.0)	29/9 (31.0)
Huacullani	Huacullani	21/11	52.4	4–90	74.0	120/81 (67.5)	210/95 (45.2)
Queroni	Huacullani	7/0	0^c^	–	–	100/70 (70.0)	–
Total		84/20	23.8^b^	3–101	28.1	920/582 (63.7)	768/148 (19.3)

a *Data from Buchon et al. (56), Grock et al. (57), Mas-Coma et al. (32, 50)*.

b *Significantly different vs. sheep and cattle, determined by Chi-square/test Chi-square with Yates' correction (p < 0.05)*.

c *Statistical comparison could not be made*.

In the Altiplano, donkeys are usually found isolated, only rarely more than one specimen accompanying grazing herds of sheep and/or cattle. The surveys were made to assure that a fecal sample of all donkeys found during the survey days could be obtained. Unfortunately, stool sample collection could not be made following the different annual seasons in a zone and, thus, no data are available to analyze the variations of prevalence throughout the different months of the year. In the Altiplano hyperendemic area, donkeys are not treated against liver fluke infection.

#### Stool Sample Preparation and Study

Only coprological methods for qualitative and quantitative analyses were carried out. Fecal samples were placed in numbered plastic bags, transported to the laboratory within the following 5 h, and maintained at 4°C until examination. From each stool sample a quantity of 4 g was sedimented twice, first with 50 ml of detergent solution (1 ml/1,000 cm^3^) after filtration and second with 50 ml water, and stained with methyl green according to a standard method (58), before examination under light microscope for *F. hepatica* eggs. A host individual was considered negative when no eggs were found in its respective stool sample after studying 3 slides. The number of eggs shed by a donkey was used to estimate the infection intensity and was expressed in eggs per gram of stools (epg).

Egg measurements were carried out using a computer image analysis system (CIAS) on the basis of standardized measurements known to be useful for fasciolid species (59). Standardized measurements were taken using a microscope and images captured by a digital camera (3CCD color videocamera Sony DXC-930P), which were then analyzed by image analysis software (ImagePro plus version 5.0 for Windows, Media Cybernetics, Silver Spring, Maryland, USA). Egg characteristics studied were: (a) linear measurements: egg length (EL), egg width (EW), and egg perimeter (EPe); (b) areas: egg area (EA); (c) ratios: EL/EW ratio. For each measure, minimum and maximum values, mean, and standard deviation were determined.

### Statistical Analyses

To assess the transmission capacity and epidemiological role of the donkey isolate, each characteristic studied was compared with that of the sheep and cattle isolates of *F. hepatica* from the same Northern Bolivian Altiplano, which have been proved to be the main animal reservoir species in this human hyperendemic area. This comparative assessment is feasible because based on data obtained following the same methods and techniques both in the laboratory experiments and in the field work and which have been reported in another study (50).

Statistical analyses were performed using SPSS Statistics 26. Development egg data (E.E.D.M., E.A.M., E.O.M., E.D.M.) were compared by ANOVA test. Statistical comparison of categorical variables was carried out with the Chi-square test and Yates continuity corrected Chi-square test. Means obtained in data from experimental infections of lymnaeid snails and from experimental infections of Wistar rats were compared by non-parametric Kruskal-Wallis test. Fasciolid egg size measurements (EL, EW, EPe, and EL/EW) were compared by *post-hoc* tests (L.S.D., Student–Newman–Keuls and Duncan's tests). Results were considered statistically significant when *p* < 0.05.

## Results

### Egg Embryonation

**Figure 3:**
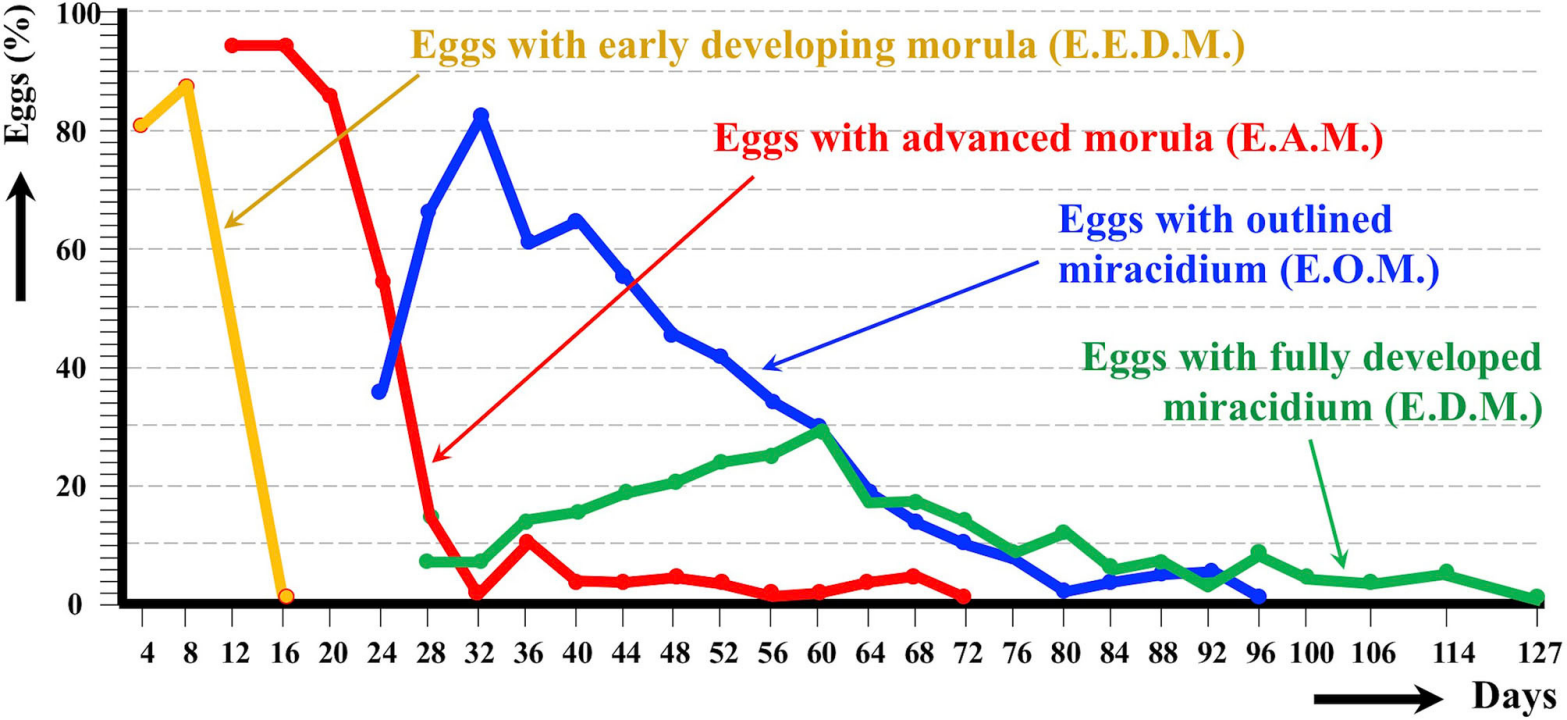
Graph showing the results of the experimental follow-up study of the egg embryonation of the altiplanic donkey isolate of *Fasciola hepatica*, at 4-day study intervals and constant temperature of 20°C. Curves of the percentages of degenerated, empty, and broken eggs are not included.

The egg embryonation of the donkey isolate of altiplanic *F. hepatica* could be experimentally followed at 20°C until complete development of all eggs (Figure 3). An outlined miracidium form begins to be observed inside eggs at day 24. The first fully developed miracidium appears at day 28. Eggs including a fully developed miracidium were henceforth observed in each observational day. The percentage of fully developed miracidia follows an increasing curve until a peak on day 60, after which it gradually decreases until day 127. No statistically significant differences in the average egg embryonation (E.O.M., E.D.M.) of the donkey isolate (36.63%) in the 60 days were detected when compared to the sheep isolate (38.93%) and cattle isolate (26.63%) from the Altiplano (*p* > 0.05).

### Snail Infectivity and Intramolluscan Development

Snail vector infection assays performed with the altiplanic donkey isolate furnished results which are shown in Table 1, including their comparison with the altiplanic sheep and cattle isolates. The donkey isolate (28.0%) was even slightly more efficient than the cattle isolate (25.0%), although both at distance from the sheep isolate (51.8%). Nevertheless, no statistically significant differences in the percentage of snails successfully infected by a monomiracidial dose at 20/20°C (= the snail infectivity) between the donkey isolate and the two main reservoirs (*p* > 0.05) were detected.

The donkey isolate prepatent period (60.5 days) showed a slightly higher mean value than the sheep isolate (55.6 days) and the cattle isolate (55.5 days). However, no statistically significant differences in prepatent period between the donkey isolate and the two main reservoirs (*p* > 0.05) were detected. The donkey isolate cercarial shedding period (average: 26.6 days) showed a shorter mean value than the sheep isolate (average: 34.7 days) and the cattle isolate (average: 47.1 days). No statistically significant differences in the cercarial shedding period were anyway detected between the donkey isolate and the two main reservoirs (*p* > 0.05).

Significant differences were observed when comparing the smaller production of cercariae per infected snail of the donkey isolate (average: 47.6 cercariae/snail) with that of the sheep (average: 197.9 cercariae/snail) and cattle (average: 306 cercariae/snail) isolates (*p* < 0.05).

The assessment of the infection impact on snails was measured by the snail survival after shedding end in days, as well as by the comparison of the longevity of shedding and non-shedding snails. These three characteristics in the donkey isolate showed mean and maximum dpi values clearly shorter than in sheep and cattle isolates, all of them proving to be statistically different (*p* < 0.05).

### Chronobiology of Cercarial Shedding

**Figure 4:**
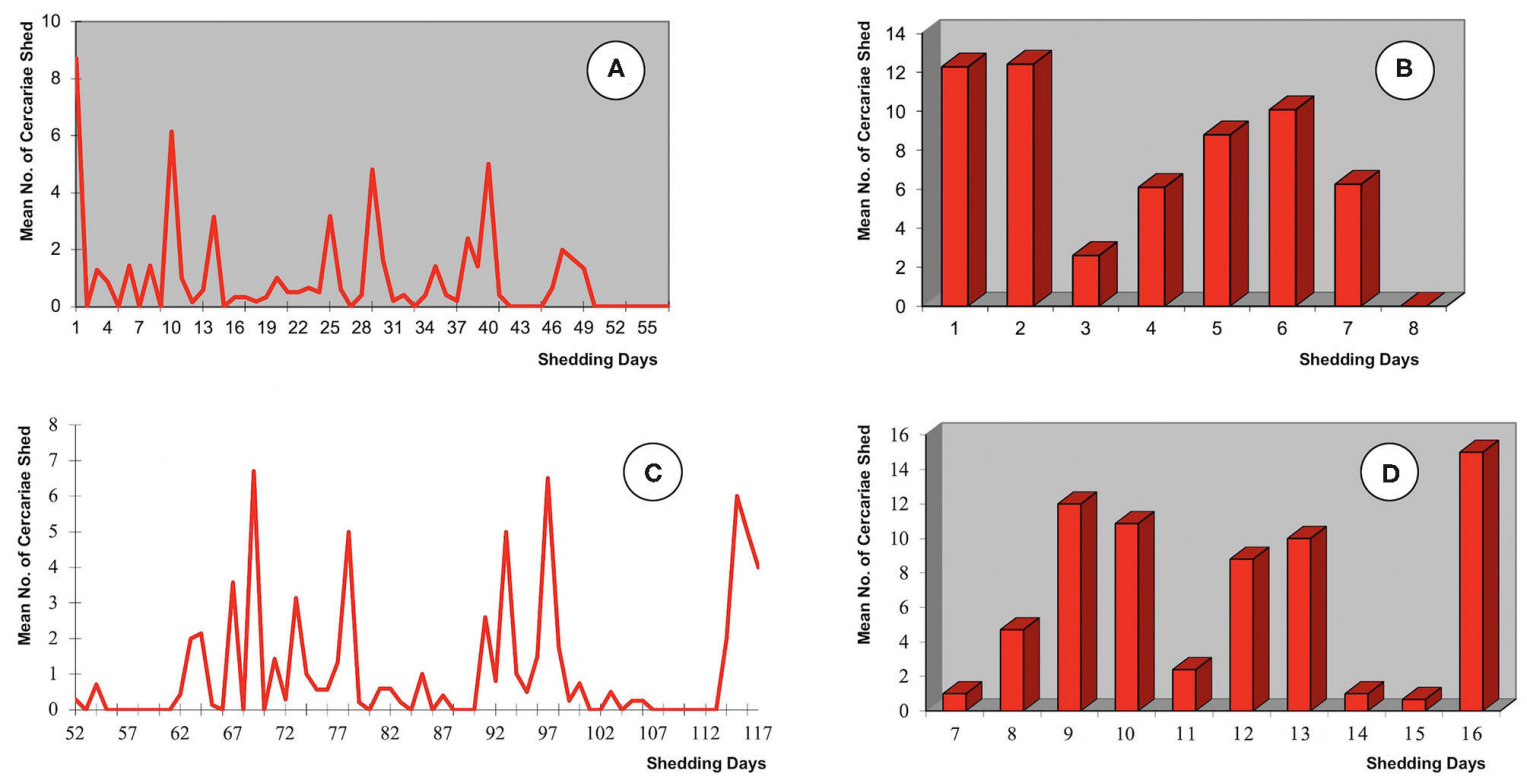
Chronobiological patterns of cercarial emergence by altiplanic *Galba truncatula* monomiracidially infected with the donkey isolate of *Fasciola hepatica* from the Northern Bolivian Altiplano human fascioliasis hyperendemic area: **(A,B)** shedding period analyzed according to the mean amounts of cercariae shed daily and weekly from the day of the emergence of the first cercaria by each snail; **(C,D)** shedding period analyzed according to the mean amounts of cercariae shed daily and weekly from the day of the miracidial infection; prepatent period not shown.

The chronobiological patterns of cercarial emergence in the donkey isolate are shown in Figures 4A–D. The shedding period analyzed according to the mean amounts of cercariae shed daily and weekly from the day of the emergence of the first cercaria by each snail is shown in Figures 4A,B, The total length of the shedding period lasted up to 49 days (Figure 4A) or 7 weeks (Figure 4B), with a mean of 26.6 days (Table 1). The daily shedding process appears as an irregular succession of waves in which up to seven peaks may be distinguished. Among them, the first peak is the highest and corresponds to the first day of shedding. The weekly analysis of the curve shows that the highest numbers of cercariae are shed during the first 2 weeks. Interestingly, a gradual increase of the weekly cercarial number from week 3 gives rise to a late peaks on week 6.

When the analysis is made from the day of the miracidial infection, the shedding period shows completely different daily and weekly curves (Figures 4C,D). When compared with the aforementioned chronobiological patterns from the day of the emergence of the first cercaria by each snail (Figures 4A,B), several similar peaks appear but two main differences may be distinguished, namely the complete disappearance of the initial peak and the appearance of an evident final peak. These initial and final differences are well-evident in both the daily and the weekly curves. Different delays in the beginning of the cercarial shedding according to different snail specimens, due to individual differences in the prepatent period (Table 1), explain the longer daily and weekly curves obtained in this analysis.

### Mammal Host Infectivity

Results of the experimental infection of Wistar rats with experimentally obtained metacercariae of the altiplanic donkey isolate are shown in Table 2. No statistically significant differences appeared in the infectivity between the donkey isolate and the sheep and cattle isolates regarding the percentage of rats successfully infected (donkey: 38.9%; sheep: 78.4%; cattle: 75.0%; *p* > 0.05). Furthermore, the intensity of infection, measured by the number of flukes recovered per metacercariae inoculated and also per rat, did not show significant differences when comparing the donkey isolate with the sheep and cattle isolates (*p* > 0.05).

### Prevalence, Intensity, Egg Measurements, and Shedding Rates

Results obtained in the coprological surveys on donkeys inhabiting selected zones of the fascioliasis hyperendemic area (Figure 1) are shown in Table 3. However, in the Altiplano the distribution of donkeys is unfortunately very dispersed because of their reduced number when compared to other domestic animals as sheep, cattle, or pigs. So, this equine most frequently appears as individuals isolated throughout large tracts of land and consequently the donkey prevalence data should be considered with the appropriate caution.

A total of 161 eggs from donkey stools were measured. Results and the comparative analysis with fasciolid eggs shed by altiplanic sheep and cattle are shown in Table 4. The statistical analysis by *post-hoc* tests (L.S.D., Student–Newman–Keuls and Duncan's tests) showed that, in the Altiplano, EL, EW, EPe, and EL/EW of the fasciolid eggs shed by donkeys are significantly different from the same parameters in the sheep and cattle isolates (*p* < 0.05).

**Table 4:** Comparison of egg measurements **(A)** and egg shedding **(B)** between the *Fasciola hepatica* donkey isolate and the sheep and cattle isolates in the Northern Bolivian Altiplano hyperendemic area.

**Host**	**Donkey**		**Sheep**		**Cattle**	
**No. of eggs studied**	**161**		**104**		**168**	
**Measurements in μm**	**Range**	**Mean ± SD**	**Range**	**Mean ± SD**	**Range**	**Mean ± SD**
**(A) Egg Measurements**						
EL	96.4–140.8	125.4 ± 8.3	114.8–151.2	130.8 ± 7.1	105.3–155.9	132.0 ± 10.5
EW	63.3–84.7	75.0 ± 3.7	65.5–81.4	72.6 ± 3.9	61.7–82.5	71.1 ± 4.4
EPe	272.1–350.4	318.8 ± 16.3	294.2–368.2	327.6 ± 15.0	270.6–422.9	340.0 ± 33.4
EA	5,562.6–8,686.2	7,177.4 ± 646.1	5,998.2–8,608.4	7,238.0 ± 532.8	5,286.5–9,676.8	7,170.2 ± 802.5
EL/EW	1.3–2.0	1.7 ± 0.1	1.5–2.1	1.8 ± 0.1	1.6–2.3	1.8 ± 0.2
**(B) Egg Shedding**						
Intensity range (epg)	3–101		3–241^a^		1–96^b^	
Stools/day (kg)	3–8^c^		1–3^d^		15–35^d^	
No.eggs/animal/day^e^	9,000–808,000		3,000–723,000		15,000–3,360,000	

*EL, egg length; EW, egg width; EPe, egg perimeter; EA, egg area; EL/EW, egg length/egg width. Measurement values shown as range and mean ± standard deviation (SD)*.

a *According to Mas-Coma et al. (49)*.

b *After Buchon et al. (56)*.

c *Deduced for altiplanic donkeys from their weight*.

d *According to various sources*.

e *Estimations of the range of the number of F. hepatica eggs faecally shed by an animal per day in the Bolivian Altiplano*.

Intensity of infection in donkeys, measured by eggs shed by gram of feces, varied between 3 and 101 epg, with a mean of 28.1 epg (Table 3). When considering the amount of stools defecated by each domestic animal species per day, the total number of *F. hepatica* eggs shed with feces by each domestic animal species per day in the Bolivian Altiplano could be estimated (Table 4). The daily egg output per donkey per day proved to fit well with the same estimations for sheep and cattle.

## Discussion

### Donkey Participation in Fascioliasis Transmission

In the life cycle of digenean trematodes, the infection and development of the adult stage in a definitive host species does not mean that this species participates in the transmission of the parasite. This definitive host species may only represent a dead end for the digenean life cycle. In several trematode species, there may even be differences in the viability of the same definitive host species, with geographical populations playing a transmission role whereas others being a blind alley. This occurs in trematode species which show different local adaptations which may be interpreted as long-term repetitive infection events evolving locally. *Schistosoma haematobium*, a human-specific species in nature, is an illustrative example. Although natural infections by *S. haematobium* have also been reported in non-human primates (60), this does not appear to have an epidemiological importance regarding the human disease. A genetically pure strain, i.e., not an hybrid, of this schistosomatid species can be experimentally forced to adapt to a laboratory rat model after long-term reinfection passages (61).

When dealing with a trematode species with a low definitive host species specificity, as is the case of *F. hepatica* or *F. gigantic*a, results of surveys on animals in nature may give rise to misinterpretations. The finding of natural infections of a mammal species allows to include this host species among the list of animals affected by the disease caused by the trematode, but does a priori not enable to epidemiologically consider it as a reservoir because it may indeed only be a dead end. This is the case of the donkey in fascioliasis. There have been several surveys on naturally-infected donkeys, but there is still no experimental study demonstrating that it is not a dead end.

To prove that a definitive host species participates in the transmission of a trematode, there is the need to (i) experimentally assess that the life cycle of the parasite is not blocked at any of the subsequent life cycle larval stages until the infection of another definitive host individual, and (ii) demonstrate that the rates of development in all these life cycle phases is sufficient as to estimate a successful transmission in nature. In *Fasciola*, this implies appropriate laboratory research on definitive host egg shedding, egg embryonation, miracidial hatching and snail vector infection, complete intramolluscan larval development until cercarial production, cercarial shedding and its characteristics, metacercarial production and definitive host infection, and final adult stage development until mature stage. The present study has included all these aspects.

### Egg Embryonation in Altiplanic Donkey Isolate

Egg embryonation is a key aspect in the assessment of the viability of a definitive host species isolate in the life cycle of fasciolids. Even despite the maturation of the adult stage of the liver fluke, eggs shed with feces of given host species, or local geographical strain of that species, fail to reach complete embryonation of the miracidium. When successful, this process is temperature-dependent.

It should be noted that storage time can influence the development of the egg. This influence was kept to a minimum by reducing the storage period to only 1 month, that is the time from the day of collection in the field until the day after arrival to the laboratory of Valencia where the experimental study was performed taking advantage of the availability of the appropriate high accuracy climate chambers.

In ruminants, the following hatching times were found in southern Europe: 56 days at 15°C, 50 days at 18°C, 27 days at 20°C, 17 days at 25°C, 22 days at 27°C, and 17.5 days at 30°C (62). Results on first hatching times proved to be different In the southern part of Chile: 101 days at 9.1°C, 80 days at 10°C, 57 days at 12.4°C, 44 days at 12.6°C, 42 days at 13.8°C, 34 days at 15.1°C, 28 days at 16°C, 30 days at 16.4°C, and 20 days at 17°C (63). A hatching time of 19–20 days was obtained for 20°C when analyzing the linear correlation between egg development and temperature (64). At this temperature, the first fully developed miracidium in eggs shed by sheep and cattle from the Northern Bolivian Altiplano appeared at day 24 (50), that is in agreement with the 19–27 day range from the data of the aforementioned studies. In the altiplanic donkey isolate, the first fully developed miracidium appears at day 28. A similar few-day delay is also observed in the curve peak of the percentage of fully developed miracidia, at day 60 in the donkey isolate whereas at day 56 in the altiplanic sheep isolate. However, fully developed miracidia continue to appear until day 127 in the donkey isolate, which is close to day 143 in the sheep isolate. Summing up, results indicate a slightly delayed embryonation but even so a similar complete egg embryonation in length.

### Miracidial Infectivity, Intramolluscan Development, and Cercarial Chronobiology

Numerous studies on *F. hepatica*/*G. truncatula* interactions have demonstrated that the processes, including from snail infection up to cercarial shedding and related lymnaeid infection survival, are highly complex and involve many different factors which underlie a large variability (65). The monomiracidial infectivity of 28.0% at 20/20°C of 12 day/12 h night photoperiod in the altiplanic donkey isolate agrees with the range of 14.0–56.8% experimentally found in different *G. truncatula* populations in France (66, 67), as well as with that of 25.0–51.8% detected in altiplanic sheep and cattle isolates (50).

At the aforementioned miracidial dose and experimental temperature, the prepatent period (from infection up to the shedding of the first cercaria) in the altiplanic sheep and cattle isolates (50) proved to be slightly longer than in *F. hepatica*/*G. truncatula* of the lowlands (64, 68, 69). The altiplanic donkey isolate shows an even longer mean prepatent period, despite its range entering within the ranges found in the ruminants.

Under the same conditions, the patent period (length of the cercarial shedding period) in the altiplanic sheep and cattle isolates (50) agreed with the respective knowledge in ruminants of the lowlands (66, 70), but its length in the altiplanic donkey isolate proved to be shorter (Table 1).

The chronobiology of the cercarial shedding of the donkey isolate follows a pattern in which a gradual progressive decrease after an initial acrophase as observed in the altiplanic sheep and cattle isolates (71) is not observed. However, the donkey isolate shedding pattern fit well to the 1–14-wave pattern, with a 4–5-wave pattern folllowed by the majority, observed in *F. hepatica*/*G. truncatula* of the lowlands under constant conditions of temperature and photoperiod (72). Despite the shorter length of the shedding process in the donkey isolate, in the curve analyzed from the day of the miracidial infection (Figure 4C) two intervals appear in which daily shedding is completely or almost completely arrested, namely in days 80–90 and 102–113. These pauses coincide with days 90, 102, and 111 and days 84 and 121 in which cercarial shedding completely stopped by all shedding snails in the sheep and cattle isolates, respectively (71). These pauses have been linked to the redial generation replication processes.

### Cercarial Production, Lymnaeid Survival, and Metacercarial Infectivity

The shorter patent period is related to the lower range and mean of the cercarial productions per snail obtained in the altiplanic donkey isolate when compared to those of not only both altiplanic sheep and cattle isolates (Table 1) (50), but also to the low productions found in *F. hepatica*/*G. truncatula* in given lowland populations, such as 120.0 cercariae/snail (73) or 91.7 cercariae/snail (74).

The snail survival after shedding end and the longevity of shedding snails in infections by the altiplanic donkey isolate are also shorter than in those obtained when infecting with the altiplanic sheep and cattle isolates (Table 1) (50), although the results confirm that this longevity is longer in infected lymnaeids from high altitude areas than the same survival period known in *F. hepatica*/*G. truncatula* in the lowlands and that this phenomenon is independent on the host isolate of *F. hepatica* (75).

The experimental infections of Wistar rats with metacercariae of the altiplanic donkey isolate allowed to verify its definitive host infectivity (Table 2). Although the prevalences were lower, the intensities fitted well in the results obtained with the altiplanic sheep and cattle isolates (50), and also in the present knowledge when dealing with short-aged metacercariae of ruminant origin (51, 76).

### Epidemiological Role of the Donkey

The local prevalence of liver fluke infection in donkeys varied pronouncedly according to localities, with values which fit in the prevalence ranges shown by sheep and cattle in the same zones (32, 50, 56, 57). Donkeys in the more remote areas, i.e., in localities of the Tambillo-Huacullani flatland corridor distant from El Alto and La Paz, showed the highest prevalence rates. A few donkeys are usually part of the livestock of Aymara families, although their numbers vary in the different zones. Donkeys are relatively numerous in all zones of the endemic area, although in numbers markedly lower than the populations of cattle, sheep, and pigs. In the Bolivian Altiplano, donkeys are frequently found in pastures besides water collections presenting *G. truncatula* populations. The aforementioned great variability of the liver fluke infection prevalences in donkeys according to localities may be due to the local environmental conditions, mainly linked to the number of freshwater collections inhabited by lymnaeid snails.

Fasciolid infection of donkeys has been reported in many countries of Asia, Africa and the Americas, usually with low prevalence although a few exceptions of high prevalence have been published. In Asia, a local prevalence of 6.6% and an intensity of 7–17 flukes/donkey and another higher 16.6% prevalence have been reported in Iran (77). A lower prevalence of 4.1% was detected in Irak (78). A 16.13% prevalence was found in donkeys of the Central Black Sea region of Turkey (79) and another of 2.6% in Ankara city (80). In Africa, low prevalences were found in several places of Egypt, including 0.37% in Assiut, 3.03% in Gharbia, 6.7% in Al-Fayoum, and 3.08% in Giza, and mid infection rates were also found in that country, such as 14.19% in Kafr El-Sheik, 17.05% in Giza, and 17.6% in Giza and Zagazig area, as previously reviewed (81). However, markedly higher prevalences have been reported in working donkeys from Ethiopia, such as 44.4% (82) and even 80.0% (83). Anyway, low prevalences of 1.5% (84) and 5.73% (85) have also been found in this country. So, the prevalence range of 8.6–52.4% in donkeys according to local zones of the Bolivian Altiplano fit well within the aforementioned data.

In the Americas, besides Bolivia, individual donkeys have been reported to be infected by the liver fluke in Mexico (86) and Argentina (87).

Intensities, measured by individual epg amount per donkey, do not differ from the ones in altiplanic sheep and cattle. The egg outputs ranged between 3 and 145 epg (mean 44.7 epg) in altiplanic sheep (49), whereas in highly infected sheep it reached an amount of 241 epg (= 1,203 eggs per 5 g) in another previous survey performed in the same endemic area (88). In altiplanic cattle, fascioliasis intensity was between 1 and 96 epg (mean 6.8 epg) (49).

Eggs of *F. hepatica* shed by naturally infected donkeys of the Bolivian Altiplano prove to be shorter but thicker than those found in naturally infected altiplanic sheep and cattle (Table 4). This agrees with present knowledge about the influence of the definitive host species on the size of fasciolid eggs (89). Interestingly, *F. hepatica* eggs found in palaeoparasitological studies of old donkeys (onagers) in the former Fertile Crescent area, at present Iran, during the Sassanid period, 224-651 AD, also fit in the egg size ranges found in present day donkeys (81).

The total number of liver fluke eggs expelled with feces by a donkey per day in the Northern Bolivian Altiplano, according to the amount of stools defecated by a donkey per day, may be estimated to be between 9,000 and 808,000 eggs/donkey/day. This range overlaps with similar estimates made for sheep and cattle in the same Northern Altiplano hyperendemic area (Table 4).

Finally, the susceptibility of the mammal host species to liver fluke infection is an important additional aspect to be considered in a One Health control initiative. If the animal is only able to survive a given period after the infection, its contribution to the disease transmission may be only for a short time and thus negligible when compared with other mammals able to transmit for the rest of their life, as is the case of sheep. If presenting a high immunological refractory response, liver flukes will survive less time inside that host and the contribution to transmission will consequently be restricted in quantity and time, as is the case of several cattle races. Fascioliasis is known to be different in equines and ruminants from the pathogenicity point of view, with the horse as the most resistant, the donkey as the most susceptible, and the mule showing intermediate profile although closer to those of the horse (90). Indeed, *F. hepatica* infection has been reported to be fatal for donkeys (86), and the pathology caused by a *F. gigantica* infection in donkeys has already been analyzed post-mortem in Egypt (91).

The lower adaptation of *F. hepatica* to the donkey is reflected in the longer prepatent period and mainly in the significantly lower number of cercariae produced by infected snails (Table 1). This is a priori not surprising, considering the phylogenetic distance between equines and ruminants. Indeed, the origin of *F. hepatica* is known to be linked to mid-sized ovicaprines (5). However, similar studies would be appropriate to assess whether the liver fluke may locally increase its adaptability to the donkey in other regions of the world, mainly eastern Africa and near-eastern Asia.

In mules, it was observed that a massive infection by 97 adult flukes was highly pathogenic and finally fatal, and that the maximum daily output was 794,500 eggs/mule/day (90). By extrapolation from mule data, the maximum of 808,000 eggs/donkey/day estimated in the Altiplano would mean a massive infection of a donkey by 99 flukes. However, amounts higher than 70 epg were only found in three donkeys, that is the 15.0% of the infected donkeys and 3.6% of the total of donkeys analyzed. Hence, massive infections representing death risk may be only sporadically reached in altiplanic donkeys and probably only in old specimens kept in a highly contaminated place for long periods.

The donkey played a crucial role in human history (92). Of African origin, it was initially domesticated in East-North Africa and the Near East Asian region (93) and was introduced into the Americas by the Spanish “conquistadores” (5). Donkeys have a range of physiological and behavioral adaptations providing them with advantages to survive extreme conditions such as those of drought and high altitude environments. They spend less energy while foraging for food which results in a lower dry matter intake requirement. Low-quality diets are digested by donkeys very efficiently because of a highly selective feeding strategy. The lower energy costs of walking, the longer foraging times per day, and their ability to tolerate thirst, allow donkeys to access more remote, under-utilized sources of forage that are inaccessible to ruminants (94). Thus, although the main introduction purpose was the *in-situ* production of mules, donkeys easily adapted to the hypoxic conditions of the high altitude environments of the Andean valleys and altiplanos, where they were increasingly used by the high altitude communities because of the lower adaptation capacity of horses (95). The biochemical and hematological parameters in mules at high altitude have recently been assessed (90), and in donkeys the respective reference intervals have also been established (96), including the Creole donkey in South America (97). However, the analysis of these parameters in high altitude for Andean donkeys is still pending. Donkeys with ligated bile ducts exhibited the biochemical, pathological, and clinical features including motor coordination impairment, constipation, oedema, dermatitis, and jaundice, together with serum increased levels of total bilirubin and ammonia and decreased levels of total protein and calcium (98).

The donkey, as equines in general, lacks a gallbladder. This means that the bile produced by the liver goes directly to the small intestine, and it is not stored. Animals lacking a gallbladder tend to eat small amounts of food several times a day. Furthermore, the natural diet of grasses that donkeys ingest while grazing is not particularly high in fat, and consequently they digest and absorb it well. Thus, from the point of view of the liver fluke transmission, the eggs cannot be stored in the gallbladder and their faecally excretion should be more regular and constant than in animals having a gallbladder.

Infection by the liver fluke may underlie severe pathogenicity in donkeys. Indeed, the high efficiency of donkeys for energy storage and mobilization has also made them prone to pathological conditions associated with negative energy balance. Pathological consequences mainly concern excessive lipolysis, excessive hepatic triglyceride synthesis, and release into systemic circulation (dyslipidemia), with severe systemic damage. Mortality rates up to 80% have been reported. Dyslipidemias in donkeys occur secondary to physiological dysfunctions and pathological processes, among which mainly liver disease (99). Donkeys are particularly susceptible to hyperlipaemia, a disease caused by too much fat in the blood. When a donkey stops eating enough it goes into a state of negative energy balance. In this state, the body begins to use energy stored as fat deposits and consequently free fatty acids are converted to glucose in the liver under a system controlled by complex hormonal events. Donkeys are not able to appropriately turn off this fat release and blood circulation soon fills up with excess fat. Large amounts of fat trigger liver and kidney failure. Subsequently, all organs may fail leading to irreversible organ damage and death (99). Detection of an increase of plasma triglyceride levels (hyperlipemia) is crucial to assess fat circulation. Measuring the levels of other serum markers indicating liver dysfunction may additionally help. The alterations of serum biochemical and hematological parameters have recently been studied in the infection by fasciolids in both the acute and chronic phase of the disease (7) and may be used for donkey control within a One Health action.

## Concluding Remarks

The experimental studies performed demonstrate that the donkey is a definitive host which assures the viability of the whole life cycle of *F. hepatica* in the Northern Bolivian Altiplano human hyperendemic area. Several aspects indicate, however, that *F. hepatica* does not reach in the donkey the level of adaptation it shows in sheep and cattle in this very high altitude area. This is illustrated by a few-day delay in egg embryonation, longer prepatent period despite similar miracidial infectivity and shorter patent period in the intramolluscan development, lower cercarial production per snail, different cercarial chronobiology, shorter snail survival after shedding end, shorter longevity of shedding snails, and lower metacercarial infectivity in Wistar rats. Thus, the role of the donkey in the transmission of the disease should be considered secondary.

Results of the field surveys proved that liver fluke prevalence and intensity in donkeys are similar to those of the main reservoirs sheep and cattle in this hyperendemic area. Worth mentioning, moreover, fasciolid egg shedding by a donkey individual proved to contribute to the environment contamination at a rate similar to sheep and cattle in that area. The very low percentage of donkeys with high infection burdens suggests a negligible liver fluke pathological impact on donkey survival and hence a long-term contribution of infected donkeys to fascioliasis transmission. In this endemic area, nevertheless, the pronounced lower number of donkeys when compared to sheep and cattle populations indicate that the epidemiological reservoir role of the donkey is also secondary when compared to these ruminants.

**Figure 5:**
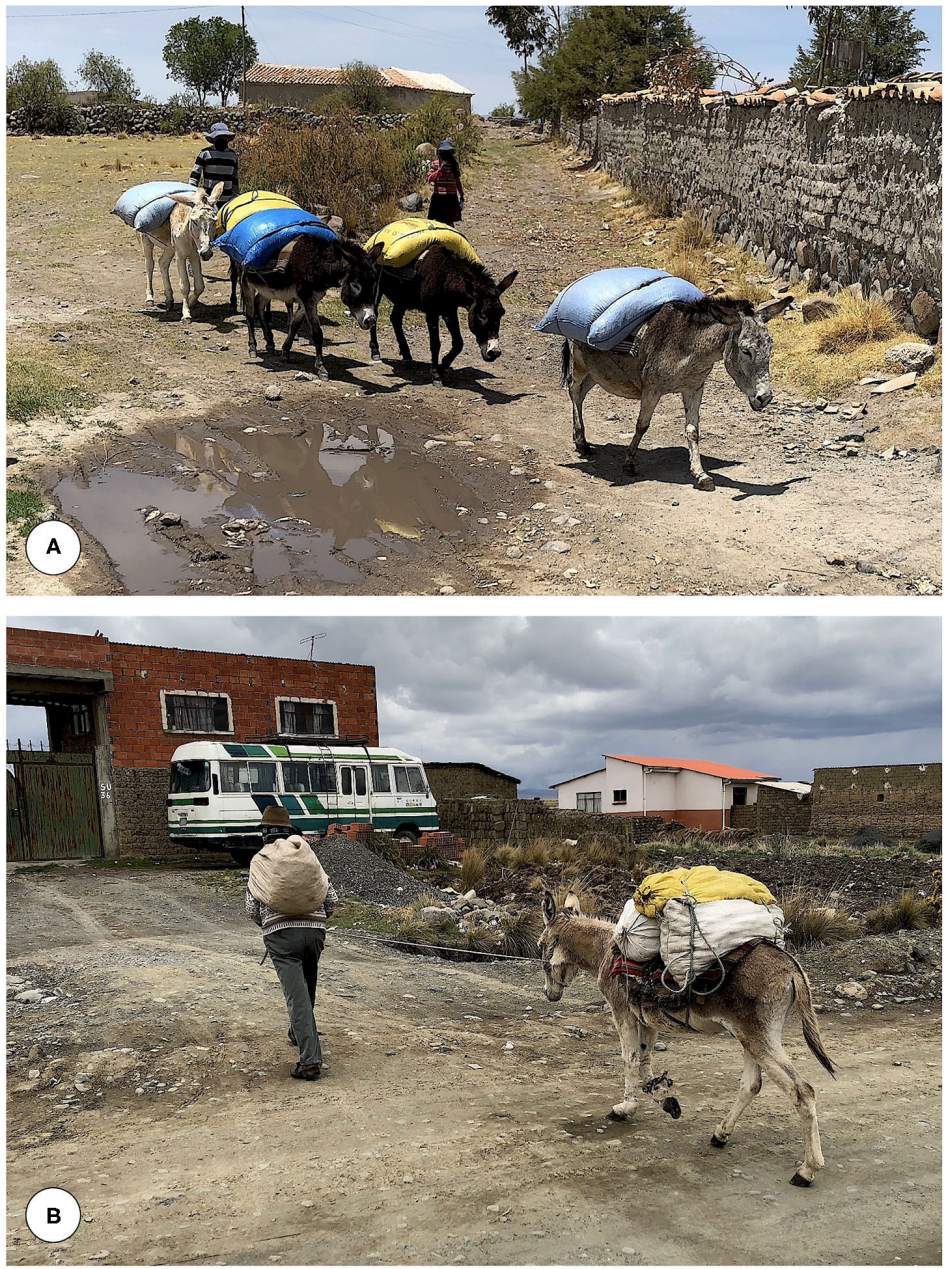
In the Northern Bolivian Altiplano human fascioliasis hyperendemic area, **(A)** donkeys are used for the transport of goods, and **(B)** for movements and travel from one locality/zone to another. Orig. S. Mas-Coma.

There is, however, an aspect of the donkey related to its management by the Aymara inhabitants which has a crucial repercussion in the transmission and epidemiology of the disease in the human hyperendemic area of the Bolivian Altiplano. Aymaras use the donkeys for the transport of goods and merchandises and for movements and travel from one locality/zone to another (Figure 5). Thus, this equine host species plays an important epidemiological role in the spread of the disease from one zone to another inside the wide endemic area. The participation of the donkey in this disease dissemination takes place in a dual manner. On one side by releasing fasciolid eggs along the routes they follow and on another side by passively transporting the lymnaeid vectors. According to what is known, lymnaeids may remain in dried mud stuck to the feet of animals, then go into hibernation or estivation, and are able to reactivate once in a new location following contact with water or sufficient humidity (5). This becomes even more important in a period as the present one in which the geographical distribution of the disease is spreading throughout the Northern Altiplano due to climate change (47).

Additionally, this fascioliasis spreading capacity of the donkey poses a problem for the implementation of a One Health initiative, because the donkey may give rise to movements of the parasite and the vector from one part to another of the zone selected for control intervention, or the introduction of the parasite and/or the vector from outside into that zone.

This study is an example of the appropriate and complete way to assess the participation of a mammal host species in the transmission and epidemiology of fascioliasis in an endemic area. It illustrates the long-term research complexity and difficulties involved in the needed experimental tasks and field activities in a disease of very high heterogeneity as that caused by liver flukes. To clarify the participation rate of a definitive host species in the case of parasites of low definitive host species specificity as fasciolids by comparing with the participation rates of other definitive host species in the same endemic area becomes crucial to establish correct control measure priorities within a One Health action. All in all, the extreme complexity of a One Health action against fascioliasis becomes evident, further if it is considered that in the Northern Bolivian Altiplano the simplicity is maximum because the disease is only caused by *F. hepatica* and transmitted by only genetically uniform *G. truncatula*, and that the present study only concerns the One Health intervention axis of the mammal reservoirs.

## Data Availability Statement

The datasets generated for this study are available on request to the corresponding author.

## Ethics Statement

All experimental research was performed with the approval of the Evaluation of Projects concerning Animal Research at University of Valencia (Organo Habilitado para la Evaluación de Proyectos de Experimentación Animal de la Universidad de Valencia) (A1263 915389140), strictly following the institution's guidelines based on Directive 2010/63/EU. Permission for animal research was additionally obtained from the Servicio de Sanidad y Bienestar Animal, Dirección General de Producción Agraria y Ganadería, Consellería de Presidencia y Agricultura, Pesca, Alimentación y Agua, Generalitat Valenciana, Valencia, Spain (No. 2015/VSC/PEA/00001 tipo 2). Animal ethics guidelines regarding animal care were strictly adhered. The study was approved by the Comisión de Etica de la Investigación of the Comité Nacional de Bioética, La Paz (Certificate dated 10 September 2007), Comité de Etica y Bioética de la Facultad de Medicina de la Universidad Mayor de San Andres, UMSA, La Paz - COMETICA (Resolución COMETICA No. 03/2019, dated 23 July 2019), and Comité de Revisión Etica (PAHOERC) of the Pan American Health Organization, PAHO, Washington DC (Dictamen Ref. No. 2018-02-0007, dated 10 September 2019). Written informed consent for participation was not obtained from the owners because all investigations were made after permission was obtained from local Aymara community chiefs (jilakatas and malkus), and with the consent of animal owners.

## Author Contributions

SM-C performed One Health studies, designed the protocols, wrote the manuscript, and obtained project funding. PB performed the surveys and diagnosis of animals. IRF performed the snail cultures, experimental snail infections, and chronobiological assessments. RA coordinated local research and logistics and participated in surveys. CM-B reviewed donkey physiology and metabolism and analyzed parasite-induced pathological repercussions. PA participated in surveys and diagnosis of animals. MAV performed the experimental mammal infections, statistical analyses, and obtained project funding. MDB performed the egg development studies, designed and participated in the experimental snail infections, participated in surveys, and obtained project funding. All authors contributed to the article and approved the submitted version.

## Conflict of Interest

The authors declare that the research was conducted in the absence of any commercial or financial relationships that could be construed as a potential conflict of interest.

## References

[1] Chen MG Mott KE Progress in assessment of morbidity due to *Fasciola hepatica* infection: a review of recent literature. Trop Dis Bull. (1990) 87:R1–38.

[2] Mas-Coma S Bargues MD Valero MA. Diagnosis of human fascioliasis by stool and blood techniques: update for the present global scenario. Parasitology. (2014) 141:1918–46. 10.1017/S0031182014000869 25077569

[3] Mas-Coma S. Human fascioliasis emergence risks in developed countries: from individual patients and small epidemics to climate and global change impacts. Enf Emerg Microbio Clí*n*. (2020) 38:253–6. 10.1016/j.eimc.2020.01.014 32107024

[4] World Health Organization Sustaining the Drive to Overcome the Global Impact of Neglected Tropical Diseases. Geneva: Department of Control of Neglected Tropical Diseases, World Health Organization, WHO Headquarters (2013). 128. p

[5] Mas-Coma S Valero MA Bargues MD. *Fasciola*, lymnaeids and human fascioliasis, with a global overview on disease transmission, epidemiology, evolutionary genetics, molecular epidemiology and control. Adv Parasitol. (2009) 69:141–6. 10.1016/S0065-308X(09)69002-3 19622408

[6] Valero MA Girones N Garcia-Bodelon MA Periago MV Chico-Calero I Khoubbane M. Anaemia in advanced chronic fasciolosis. Acta Trop. (2008) 108:35–43. 10.1016/j.actatropica.2008.08.007 18805388

[7] Valero MA Bargues MD Khoubbane M Artigas P Quesada C Berinde L. Higher physiopathogenicity by *Fasciola gigantica* than by the genetically close *F. hepatica*: experimental long-term follow-up of biochemical markers. Trans Roy Soc Trop Med Hyg. (2016) 110:55–66. 10.1093/trstmh/trv110 26740363

[8] Mas-Coma S Agramunt VH Valero MA. Neurological and ocular fascioliasis in humans. Adv Parasitol. (2014) 84:27–149. 10.1016/B978-0-12-800099-1.00002-8 24480313

[9] Dalton JP Robinson MW Mulcahy G O'Neill SM Donnelly S. Immunomodulatory molecules of *Fasciola hepatica*: candidates for both vaccine and immunotherapeutic development. Vet Parasitol. (2013) 195:272–85. 10.1016/j.vetpar.2013.04.008 23623183

[10] Girones N Valero MA Garcia-Bodelon MA Chico-Calero MI Punzon C Fresno M. Immune supression in advanced chronic fascioliasis: an experimental study in a rat model. J Infect Dis. (2007) 195:1504–12. 10.1086/514822 17436231

[11] O'Neill SM Brady MT Callanan JJ Mulcahy G Joyce P Mills KH. *Fasciola hepatica* infection downregulates Th1 responses in mice. Parasite Immunol. (2000) 22:147–55. 10.1046/j.1365-3024.2000.00290.x 10672196

[12] Valero MA Navarro M Garcia-Bodelon MA Marcilla A Morales M Garcia JE. High risk of bacterobilia in advanced experimental chronic fasciolosis. Acta Trop. (2006) 100:17–23. 10.1016/j.actatropica.2006.09.002 17064656

[13] Esteban JG Flores A Angles R Strauss W Aguirre C Mas-Coma S. A population-based coprological study of human fascioliasis in a hyperendemic area of the bolivian altiplano. Trop Med Int Health. (1997) 2:695–99. 10.1046/j.1365-3156.1997.d01-356.x 9270738

[14] Gonzalez LC Esteban JG Bargues MD Valero MA Ortiz P Naquira C. Hyperendemic human fascioliasis in andean valleys: an altitudinal transect analysis in children of cajamarca province, Peru. Acta Trop. (2011) 120:119–29. 10.1016/j.actatropica.2011.07.002 21767521

[15] Ollerenshaw CB The ecology of the liver fluke (*Fasciola hepatica*). Vet Rec. (1959) 71:957–65.

[16] Afshan K Fortes-Lima CA Artigas P Valero MA Qayyum M Mas-Coma S. Impact of climate change and man-made irrigation systems on the transmission risk, long-term trend and seasonality of human and animal fascioliasis in Pakistan. Geospat Health. (2014) 8:317–34. 10.4081/gh.2014.22 24893010

[17] Valero MA Perez-Crespo I Chillon-Marinas C Khoubbane M Quesada C Reguera-Gomez M *Fasciola hepatica* reinfection potentiates a mixed Th1/Th2/Th17/Treg response and correlates with the clinical phenotypes of anemia. PLoS ONE. (2017) 12:e0173456 10.1371/journal.pone.0173456 28362822 PMC5376296

[18] Valero MA Girones N Reguera-Gomez M Perez-Crespo I Lopez-Garcia MP Quesada C. Impact of fascioliasis reinfection on *Fasciola hepatica* egg shedding: relationship with the immune-regulatory response. Acta Trop. (2020) 209:105518. 10.1016/j.actatropica.2020.105518 32371223

[19] Gonzalez-Miguel J Valero MA Reguera-Gomez M Mas-Bargues C Bargues MD Simon-Martin F. Numerous *Fasciola* plasminogen-binding proteins may underlie blood-brain barrier leakage and explain neurological disorder complexity and heterogeneity in the acute and chronic phases of human fascioliasis. Parasitology. (2019) 146:284–98. 10.1017/S0031182018001464 30246668 PMC6402360

[20] Rondelaud D Dreyfuss G Vignoles P. Clinical and biological abnormalities in patients after fasciolosis treatment. Med Mal Infect. (2006) 36:466–8. 10.1016/j.medmal.2006.07.018 17030486

[21] Gandhi P Schmitt EK Chen CW Samantray S Venishetty VK Hughes D. Triclabendazole in the treatment of human fascioliasis: a review. Trans Roy Soc Trop Med Hyg. (2019) 113:797–804. 10.1093/trstmh/trz093 31638149 PMC6906998

[22] World Health Organization Ending the Neglect to Attain the Sustainable Development Goals. A Road Map for Neglected Tropical Diseases 2021-2030. Geneva: World Health Organization, WHO Headquarters (2020) 47 p. Available online at: https://www.who.int/neglected_diseases/Ending-the-neglect-to-attain-the-SDGs–NTD-Roadmap.pdf

[23] Esteban JG Gonzalez C Bargues MD Angles R Sanchez C Naquira C. High fascioliasis infection in children linked to a man-made irrigation zone in Peru. Trop Med Int Health. (2002) 7:339–48. 10.1046/j.1365-3156.2002.00870.x 11952950

[24] Espinoza JR Maco V Marcos L Saez S Neyra V Terashima A. Evaluation of Fas2-ELISA for the serological detection of *Fasciola hepatica* infection in humans. Am J Trop Med Hyg. (2007) 76:977–82. 10.4269/ajtmh.2007.76.977 17488926

[25] Hillyer GV Soler de Galanes M Rodriguez-Perez J Bjorland J Silva de Lagrava M Ramirez Guzman S. Use of the Falcon Assay Screening Test - Enzyme-Linked Immunosorbent Assay (FAST-ELISA) and the Enzyme-Linked Immunoelectrotransfer Blot (EITB) to determine the prevalence of human fascioliasis in the bolivian altiplano. Am J Trop Med Hyg. (1992) 46:603–9. 10.4269/ajtmh.1992.46.603 1599055

[26] Bjorland J Bryan RT Strauss W Hillyer GV McAuley JB. An outbreak of acute fascioliasis among aymara Indians in the bolivian altiplano. Clin Inf Dis. (1995) 21:1228–33. 10.1093/clinids/21.5.1228 8589147

[27] Malandrini JB Carnevale S Velazquez J Soria CC Diagnóstico de *Fasciola hepatica* con la técnica de ELISA en el departamento de tinogasta. Ciencia. (2009) 4:143–51.

[28] Mera y Sierra R Agramunt VH Cuervo P Mas-Coma S. Human fascioliasis in Argentina: retrospective overview, critical analysis and baseline for future research. Parasit Vectors. (2011) 4:104. 10.1186/1756-3305-4-104 21663691 PMC3141741

[29] Bargues MD Malandrini JB Artigas P Soria CC Velasquez JN Carnevale S. Human fascioliasis endemic areas in Argentina: multigene characterisation of the lymnaeid vectors and climatic-environmental assessment of the transmission pattern. Parasit Vectors. (2016) 9:306. 10.1186/s13071-016-1589-z 27229862 PMC4882814

[30] Apt W Aguilera X Vega F Zulantay I Retamal C Apt P Fascioliasis en la población rural de las provincias de curico, talca y linares. Rev Méd Chile. (1992) 120:621–6. 1341790

[31] Artigas P Bargues MD Mera y Sierra R Agramunt VH Mas-Coma S. Characterisation of fascioliasis lymnaeid intermediate hosts from Chile by DNA sequencing, with emphasis on *Lymnaea viator* and *Galba truncatula*. Acta Trop. (2011) 120:245–57. 10.1016/j.actatropica.2011.09.002 21933653

[32] Mas-Coma S Angles R Esteban JG Bargues MD Buchon P Franken M. The northern bolivian altiplano: a region highly endemic for human fascioliasis. Trop Med Int Health. (1999) 4:45467. 10.1046/j.1365-3156.1999.00418.x 10444322

[33] De NV Le TH Agramunt VH Mas-Coma S. Early postnatal and preschool age infection by *Fasciola* spp.: report of five cases from Vietnam and worldwide review. Am J Trop Med Hyg. (2020) 103:1578–89. 10.4269/ajtmh.20-0139 32618259 PMC7543854

[34] Villegas F Angles R Barrientos R Barrios G Valero MA Hamed K. Administration of triclabendazole is safe and effective in controlling fascioliasis in an endemic community of the Bolivian Altiplano. PLoS Negl Trop Dis. (2012) 6:e1720. 10.1371/journal.pntd.0001720 22880138 PMC3413701

[35] Valero MA Periago MV Perez-Crespo I Angles R Villegas F Aguirre C. Field evaluation of a coproantigen detection test for fascioliasis diagnosis and surveillance in human hyperendemic areas of Andean countries. PLoS Negl Trop Dis. (2012) 6:e1812. 10.1371/journal.pntd.0001812 23029575 PMC3441400

[36] Mas-Coma S Bargues MD Valero MA. Human fascioliasis infection sources, their diversity, incidence factors, analytical methods and prevention measures. Parasitology. (2018) 145:1665–99. 10.1017/S0031182018000914 29991363

[37] Ueno H Arandia R Morales G Medina G. Fascioliasis of livestock and snail host for *Fasciola* in the altiplano region of Bolivia. Nat Inst Anim Health Q. (1975) 15:61–7. 1182037

[38] Essack SY. Environment: the neglected component of the one health triad. Lancet. (2018) 2:e238–39. 10.1016/S2542-5196(18)30124-4 29880152 PMC7129022

[39] Lubroth J. FAO and the one health approach. Curr Top Microbiol Immunol. (2013) 366:65–72. 10.1007/978-3-662-45791-7_262 22949033

[40] World Health Organization Food and Agriculture Organization of the United Nations World Organisation for Animal Health. Taking a Multisectoral, One Health Approach: A Tripartite Guide to Addressing Zoonotic Diseases in Countries. FAO - OIE - WHO (2019). 151 p. Available online at: https://apps.who.int/iris/handle/10665/325620

[41] Rinaldi L Gonzalez S Guerrero J Carol Aguilera L Musella V Genchi C. A one-health integrated approach to control fascioliasis in the cajamarca valley of Peru. Geospat Health. (2012) 6:S67–73. 10.4081/gh.2012.124 23032285

[42] Webster JP Gower CM Knowles SCL Molyneux DH Fenton A. One health – an ecological and evolutionary framework for tackling neglected zoonotic diseases. Evol Appl. (2016) 9:313–33. 10.1111/eva.12341 26834828 PMC4721077

[43] Destoumieux-Garzón D Mavingui P Boetsch G Boissier J Darriet F Duboz P. The one health concept: 10 years old and a long road ahead. Front Vet Sci. (2018) 5:14. 10.3389/fvets.2018.00014 29484301 PMC5816263

[44] Bargues MD Mas-Coma S. Reviewing lymnaeid vectors of fascioliasis by ribosomal DNA sequence analyses. J Helminthol. (2005) 7 9:257–67. 10.1079/JOH2005297 16153320

[45] Bargues MD Artigas P Mera y Sierra R Pointier JP Mas-Coma S. Characterisation of *Lymnaea cubensis, L*. viatrix and *L. neotropica n. sp*., the main vectors of *Fasciola hepatica* in Latin America, by analysis of their ribosomal and mitochondrial DNA. Ann Trop Med Parasitol. (2007) 101:621–41. 10.1179/136485907X229077 17877881

[46] Bargues MD Artigas P Khoubbane M Flores R Glöer P Rojas-Garcia R. *Lymnaea schirazensis*, an overlooked snail distorting fascioliasis data: genotype, phenotype, ecology, worldwide spread, susceptibility, applicability. PLoS ONE. (2011) 6:e24567. 10.1371/journal.pone.0024567 21980347 PMC3183092

[47] Bargues MD Artigas P Angles R Osca D Duran P Buchon P. Genetic uniformity, geographical spread and anthropogenic habitat modifications of lymnaeid vectors found in a one health initiative in the highest human fascioliasis hyperendemic of the Bolivian Altiplano. Parasit Vectors. (2020) 13:171. 10.1186/s13071-020-04045-x 32252808 PMC7137187

[48] Fuentes MV Coello JR Bargues MD Valero MA Esteban JG Fons R Small mammals (Lagomorpha and Rodentia) and fascioliasis transmission in the Northern Bolivian Altiplano endemic zone. Res Rev Parasitol. (1997) 57:115–21.

[49] Mas-Coma S Rodriguez A Bargues MD Valero MA Coello JR Angles R Secondary reservoir role of domestic animals other than sheep and cattle in fascioliasis transmission in the Northern Bolivian Altiplano. Res Rev Parasitol. (1997) 57:39–46.

[50] Mas-Coma S Buchon P Funatsu IK Angles R Artigas P Valero MA Sheep and cattle reservoirs in the highest human fascioliasis hyperendemic area: experimental transmission capacity, field epidemiology and control within a one health initiative in Bolivia. Front Vet Sci. (in press). 10.3389/fvets.2020.583204 PMC7655135 33195605

[51] Boray JC Enigk K. Laboratory studies on the survival and infectiivity of Fa*sciola hepatica* and *F. gigantica* metacercariae. Z Tropenmed Parasitol. (1964) 15:324–31. 14316630

[52] Bargues MD Gayo V Sanchis J Artigas P Khoubbane M Birriel S. DNA multigene characterization of *Fasciola hepatica* and *Lymnaea neotropica* and its fascioliasis transmission capacity in Uruguay, with historical correlation, human report review and infection risk analysis. PLoS Negl Trop Dis. (2017) 11:e0005352. 10.1371/journal.pntd.0005352 28158188 PMC5310921

[53] Valero MA Panova M Comes AM Fons R Mas-Coma S. Patterns in size and shedding of *Fasciola hepatica* eggs by naturally and experimentally infected murid rodents. J Parasitol. (2002) 88:308–13. 10.1645/0022-3395.2002.088[0308:PISASO]2.0.CO;2 12054003

[54] Valero MA Mas-Coma S. Comparative infectivity of *Fasciola hepatica* metacercariae from isolates of the main and secondary reservoir animal host species in the Bolivian Altiplano high human endemic region. Folia Parasitol. (2000) 47:17–22. 10.14411/fp.2000.004 10833011

[55] Valero MA Santana M Morales M Hernandez JL Mas-Coma S. Risk of gallstone disease in advanced chronic phase of fascioliasis: an experimental study in a rat model. J Infect Dis. (2003) 188:787–93. 10.1086/377281 12934197

[56] Buchon P Cuenca H Quiton A Camacho AM Mas-Coma S Fascioliasis in cattle in the human high endemic region of the Bolivian Northern Altiplano. Res Rev Parasitol. (1997) 57:71–83.

[57] Grock R Morales G Vaca JL Mas-Coma S Fascioliasis in sheep in the human high endemic region of the Northern Bolivian Altiplano. Res Rev Parasitol. (1998) 58:95–101.

[58] Dennis WR Stone WM Swanson LE. A new laboratory and field diagnostic test for fluke ova in feces. J Amer Vet Med Ass. (1954) 124:47–50. 13117747

[59] Valero MA Panova M Mas-Coma S. Phenotypic analysis of adults and eggs of *Fasciola hepatica* by computer image analysis system. J Helminthol. (2005) 79:217–25. 10.1079/JOH2005301 16153315

[60] Pitchford RJ. A check list of definitive hosts exhibiting evidence of the genus *Schistosoma* Weinland, 1858 acquired naturally in Africa and the Middle East. J Helminthol. (1977) 51:229–51. 10.1017/S0022149X00007574 340501

[61] Gear JHS Davis DHS Pitchford RJ. The susceptibility of rodents to schistosome infection, with special reference to *Schistosoma haematobium*. Bull World Health Organ. (1966) 35:213–21. 5297005 PMC2476116

[62] Diez-Baños MA Rojo-Vázquez FA Influencia de la temperatura en el desarrollo de los huevos de *Fasciola hepatica*. An Fac Vet León. (1976) 22:65–75.

[63] Valenzuela G. Estudio epidemiológico acerca del desarrollo de huevos de *Fasciola hepatica* en el medio ambiente en Valdivia, Chile. Bol Chil Parasit. (1979) 34:31–5. 540082

[64] Wilson RA Smith G Thomas MR Fascioliasis. In: Anderson RM Editor. The Population Dynamics of Infectious Diseases: Theory and Applications. London; New York, NY: Chapman and Hall (1982). p. 262–319. 10.1007/978-1-4899-2901-3_9

[65] Kendall SB Nutritional factors affecting the rate of development of *Fasciola hepatica* in *Limnaea truncatula*. J Helminthol. (1993) 23:179–90. 10.1017/S0022149X00032491 15409355

[66] Rondelaud D Variabilité interpopulationelle de l'infestation fasciolenne chez le mollusque *Lymnaea truncatula* Müller. influence du contact préalable de la population avec le parasite. Bull Soc Zool France. (1993) 118:185–93.

[67] Vignoles P Dreyfuss G Rondelaud D. Larval development of *Fasciola hepatica* in experimental infections: variations with populations of *Lymnaea truncatula*. J Helminthol. (2002) 76:179–83. 10.1079/JOH2002112 12015832

[68] Roberts WE. Studies on the life-cycle of *Fasciola hepatica* (Linnaeus) and of its snail host *Limnaea* (*Galba*) *truncatula* (Müller) in the field and under controlled conditions in the laboratory. Ann Trop Med Parasitol. (1950) 44:187–206. 10.1080/00034983.1950.11685441 24538002

[69] Rondelaud D Barthe D Les générations rédiennes de *Fasciola hepatica L. Premières* observations chez des Limnées tronquées en fin de cycle parasitaire. Bull Soc Franç Parasitol. (1986) 4:29–38.

[70] Rondelaud D Dreyfuss G *Fasciola hepatica*: the influence of the definitive host on the characteristics of the infection in the snail *Lymnaea truncatula*. Parasite. (1995) 2:275–80. 10.1051/parasite/1995023275

[71] Mas-Coma S Funatsu IR Bargues MD. *Fasciola hepatica* and lymnaeid snails occurring at very high altitude in South America. Parasitology. (2001) 123:S115–27. 10.1017/S0031182001008034 11769277

[72] Dreyfuss G Rondelaud D. *Fasciola hepatica*: a study of the shedding of cercariae from *Lymnaea truncatula* raised under constant conditions of temperature and photoperiod. Parasite. (1994) 4:401–4. 10.1051/parasite/1994014401 9140507

[73] Hodasi JKM. The output of cercariae of *Fasciola hepatica* by *Lymnaea truncatula* and the distribution of metacercariae on grass. Parasitology. (1972) 63:431–56. 5010460 10.1017/s0031182000044644

[74] Audousset JC Rondelaud D Dreyfuss G Vareille-Morel C Les émissions cercariennes de *Fasciola hepatica* L. *chez* le mollusque Lymnaea truncatula Müller. A propos de quelques observations chronobiologiques. Bull Soc Franç Parasitol. (1989) 7:217–24.

[75] Bargues MD Oviedo JA Funatsu IR Rodriguez A Mas-Coma S Survival of lymnaeid snails from the Bolivian Northern Altiplano after the parasitation by different Bolivian isolates of *Fasciola hepatica* (Linnaeus, 1758) (Trematoda: Fasciolidae), In: Guerra A Rolán E Rocha F editors. Unitas Malacologica. Vigo: Instituto de Investigaciones Marinas, CSIC, (1995). p. 443–5.

[76] Kimura S Shimizu A. Studies on the survival and infectivity of *Fasciola gigantica* metacercariae. Sci Rept Fac Agr Kobe Univ. (1979) 13:347–49.14316630

[77] Hosseini SH Mesghi B Eslami A Bokai S Sobhani M Samani RE Prevalence and biodiversity of helminth parasites in donkeys (*Equus asinus*) in Iran. Int J Vet Res. (2009) 3:95–9. Available online at: https://ijvm.ut.ac.ir/article_20633_8927af20c4f9b5b2767176762bc5c686.pdf

[78] Atia AH Prevalence of *Fasciola* sp. infection in donkeys in Baghdad, Iraq. AlTaqani. (2008) 21:173–8. Available online at: https://www.iasj.net/iasj?func=article&aId=38607

[79] Umur S Acici M A survey on helminth infections of equines in the central black sea region, Turkey. Turk J Vet Anim Sci. (2009) 33:373–8. 10.3906/vet-0712-6

[80] Soykan E Öge H. The prevalence of liver trematodes in equines in different cities of Turkey. Turkiye Parazitol Derg. (2012) 36:152–5. 10.5152/tpd.2012.36 23169158

[81] Askari Z Mas-Coma S Bouwman AS Boenke N Stöllner T Aali A. *Fasciola hepatica* eggs in paleofaeces of the persian onager *Equus hemionus onager*, a donkey from chehrabad archaeological site, dating back to the sassanid empire (224-651 AD), in ancient Iran. Infect Genet Evol. (2018) 62:233–43. 10.1016/j.meegid.2018.04.028 29698771

[82] Getachew M Innocent GT Trawford AF Reid SWJ Love S. Epidemiological features of fasciolosis in working donkeys in Ethiopia. Vet Parasitol. (2010) 169:335–9. 10.1016/j.vetpar.2010.01.007 20138432

[83] Getachew M Trawford A Feseha G Reid SWJ. Gastrointestinal parasites of working donkeys of Ethiopia. Trop Anim Health Prod. (2010) 42:27–33. 10.1007/s11250-009-9381-0 19548106

[84] Ayele G Feseha G Bojia E Joe A Prevalence of gastro-intestinal parasites of donkeys in Dugda Bora District, Ethiopia. Livest. Res. Rural. Dev. (2006) 18:14–21.

[85] Mezgebu T Tafess K Tamiru F Prevalence of gastrointestinal parasites of horses and donkeys in and around Gondar Town, Ethiopia. Open J Vet Med. (2013) 3:267–72. 10.4236/ojvm.2013.36043

[86] Collins DR. Fascioliasis in Mexican burro. J Am Vet Med Assoc. (1961) 139:1321–3. 13880585

[87] Mera y Sierra RL Artigas P Cuervo P Deis E Sidoti L Mas-Coma. Fascioliasis transmission by *Lymnaea neotropica* confirmed by nuclear rDNA and mtDNA sequencing in Argentina. Vet Parasitol. (2009) 166:73–9. 10.1016/j.vetpar.2009.08.001 19729246

[88] Mas-Coma S Angles R Strauss W Esteban JG Oviedo JA Buchon P Human fascioliasis in Bolivia: a general analysis and a critical review of existing data. Res Rev Parasitol. (1995) 55:73–93

[89] Valero MA Darce NA Panova M Mas-Coma S. Relationships between host species and morphometric patterns in *Fasciola hepatica* adults and eggs from the Northern Bolivian Altiplano hyperendemic region. Vet Parasitol. (2001) 102:85–100. 10.1016/S0304-4017(01)00499-X 11705655

[90] Mera y Sierra R Neira G Bargues MD Cuervo PF Artigas P Logarzo L. Equines as reservoirs of human fascioliasis: transmission capacity, epidemiology and pathogenicity in *Fasciola hepatica* infected mules. J Helminthol. (2020) 94:e189. 10.1017/S0022149X20000693 32907643

[91] Fahmy MFM El-Attar SR Pathological study on fascioliasis in camel and solipeds. Egypt J Com Path Clinic Pathol. (1990) 3:285–91.

[92] Mitchell P The Donkey in Human History. An Archaelogical Perspective. Oxford: Oxford University Press (2018). 306. p

[93] Rossel S Marshall F Peters J Pilgram T Adams MD O'Connor D. Domestication of the donkey: timing, processes, and indicators. Proc Natl Acad Sci USA. (2007) 105:3715–20. 10.1073/pnas.0709692105 18332433 PMC2268817

[94] Smith DG Pearson RA. A review of the factors affecting the survival of donkeys in semiarid regions of Sub-Saharan Africa. Trop Anim Health Prod. (2005) 37:1–19. 10.1007/s11250-005-9002-5 16335068

[95] Wickler SJ Greene HM The horse and high altitude. Clin Tech Equine Pract. (2003) 2:231–7. 10.1053/S1534-7516(03)00066-0

[96] Burden FA Hazell-Smith E Mulugeta G Patrick V Trawford R Brooks Brownlie HW Reference intervals for biochemical and haematological parameters in mature domestic donkeys (*Equus asinus*) in the UK. Equine Vet Educ. (2016) 28:134–9. 10.1111/eve.12512

[97] Herrera BY Rugeles PC Vergara GO Perfil hematológico del burro criollo (*Equus asinus*) colombiano. Rev Colombiana Cienc Anim. (2017) 9:158–63. 10.24188/recia.v9.n2.2017.553

[98] Madany MOK Adam SEI. Ligation of the bile duct and acute chloroform toxicity in the donkey. J Comp Pathol. (1976) 86:539–46. 10.1016/0021-9975(76)90063-3 993379

[99] Mendoza FJ Toribio RE Perez-Ecija A Donkey internal medicine - Part I: metabolic, endocrine, and alimentary tract disturbances. J Equine Vet Sci. (2018) 65:66–74. 10.1016/j.jevs.2018.02.001

